# Intricate coupling between the transactivation and basic-leucine zipper domains governs phosphorylation of transcription factor ATF4 by casein kinase 2

**DOI:** 10.1016/j.jbc.2022.101633

**Published:** 2022-01-22

**Authors:** Steven Siang, Eric S. Underbakke, Julien Roche

**Affiliations:** Roy J. Carver Department of Biochemistry, Biophysics and Molecular Biology, Iowa State University, Ames, Iowa, USA

**Keywords:** transcription factor, intrinsically disordered protein, protein kinase, solution NMR spectroscopy, ATF4, activating transcription factor 4, bZip, basic-leucine zipper domain, CD, circular dichroism, CREB-2, cAMP response element-binding protein-2, HSQC, heteronuclear single quantum correlation, LC-ESI-MS, high-performance liquid chromatography coupled to electrospray ionization mass spectrometry, LC-MS-MS, liquid chromatography with tandem mass spectrometry, MALS, multiangle light scattering, NMR, nuclear magnetic resonance, SAXS, small-angle X-ray scattering, SEC, size-exclusion chromatography, TAD, transactivation domain, TROSY, transverse relaxation optimized spectroscopy

## Abstract

Most transcription factors possess at least one long intrinsically disordered transactivation domain that binds to a variety of coactivators and corepressors and plays a key role in modulating the transcriptional activity. Despite the crucial importance of these domains, the structural and functional basis of transactivation remains poorly understood. Here, we focused on activating transcription factor 4 (ATF4)/cAMP response element-binding protein-2, an essential transcription factor for cellular stress adaptation. Bioinformatic sequence analysis of the ATF4 transactivation domain sequence revealed that the first 125 amino acids have noticeably less propensity for structural disorder than the rest of the domain. Using solution nuclear magnetic resonance spectroscopy complemented by a range of biophysical methods, we found that the isolated transactivation domain is predominantly yet not fully disordered in solution. We also observed that a short motif at the N-terminus of the transactivation domain has a high helical propensity. Importantly, we found that the N-terminal region of the transactivation domain is involved in transient long-range interactions with the basic-leucine zipper domain involved in DNA binding. Finally, *in vitro* phosphorylation assays with the casein kinase 2 show that the presence of the basic-leucine zipper domain is required for phosphorylation of the transactivation domain. This study uncovers the intricate coupling existing between the transactivation and basic-leucine zipper domains of ATF4, highlighting its potential regulatory significance.

The activating transcription factor 4 (ATF4), also called cAMP response element-binding protein-2 (CREB-2), is a transcription factor that mediates cellular gene expression changes in response to different types of cellular stress (1, 2, 3). While constitutively expressed at low concentrations, ATF4 can be rapidly induced under particular cell-stress conditions such as endoplasmic reticulum stress (4), amino acid deprivation (5), and oxidative stress (6). ATF4 expression is also found to be upregulated in response to cancer progression, making it an interesting therapeutic target for blocking angiogenesis and adaptation of cancer cells to hypoxia/anoxia (7). Activity of ATF4 as a transcriptional repressor includes repression of genes encoding encephalin, chromogranin (8), and the phosphodiesterase PDE4D9 (9). ATF4 is known to form heterodimers with other transcription factors such as ATF3 (10), C/EBP homologous protein (CHOP) (11), and HTLV1 transactivator Tax (12). The numerous dimerization and interaction partners of ATF4 determine its wide range of transcriptional activities.

## ATF4 general domain architecture

**Figure 1:**
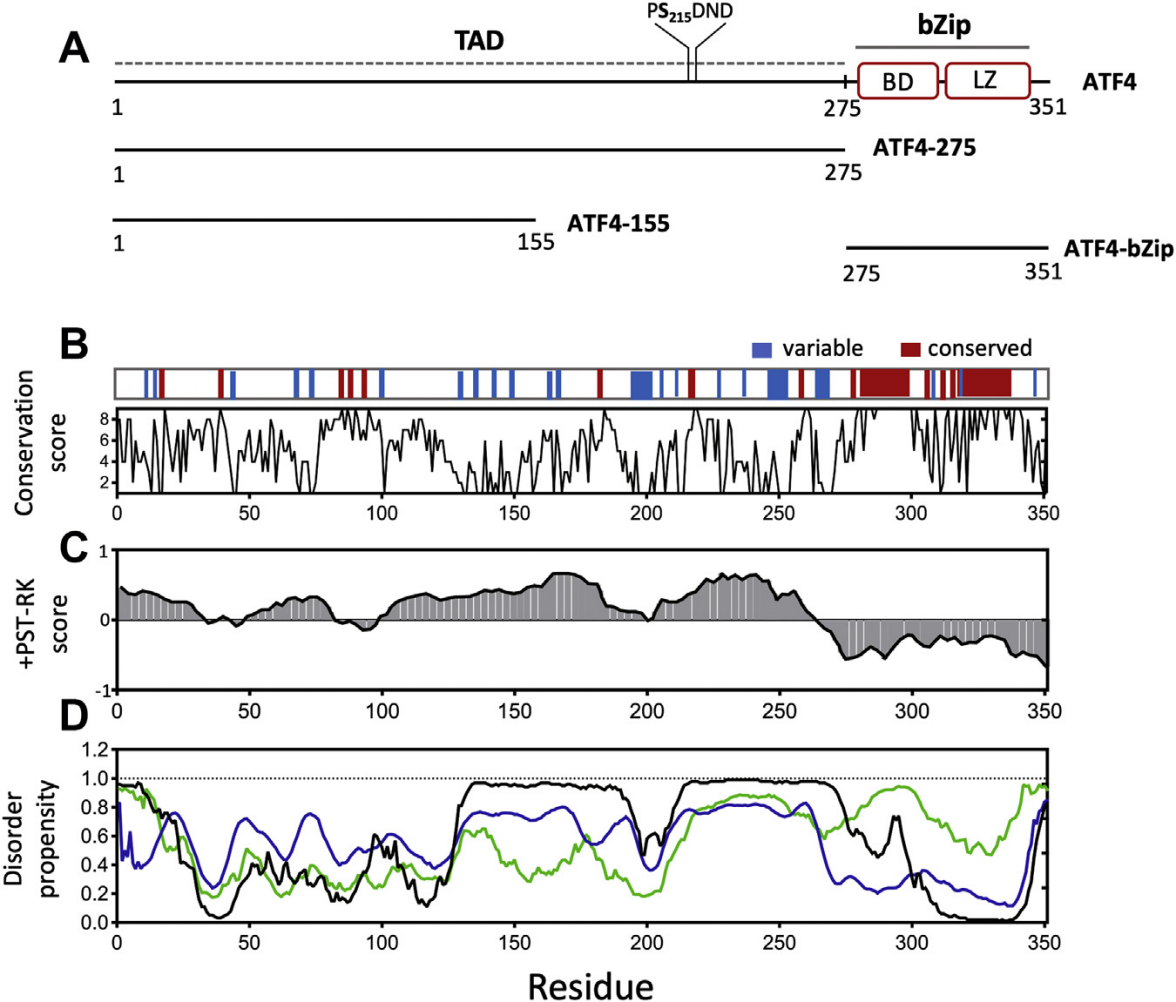
**Sequence analysis of ATF4.***A*, schematic representation of ATF4 sequence showing the N-terminal transactivation domain (TAD, residue 1–275) and C-terminal bZip region encompassing the basic domain (BD, residues 276–301) and leucine zipper domain (LZ, residues 302–351). The primary phosphorylation site of CK2, S215, is also indicated. The three additional constructs used in this study are shown below: ATF4-275 (isolated TAD), ATF4-155 (N-terminal region of the TAD), and ATF4-bZip (isolated bZip domain). *B*, conservation scores established from a Clustal W comparison of human ATF4 with five other species. Highly conserved amino acids (score > 8) and poorly conserved amino acids (score < 2) are shown in *red* and *blue*, respectively, in the *top panel*. *C*, analysis of amino acid composition with IDDomainSpotter shows regions depleted (+PST-RK score < 0) and enriched (+PST-RK score > 0) in disorder promoting amino acids. *D*, disorder propensity predicted with DisEMBL (*blue*), PONDR-FIT (*green*), and DISOPRED (*black*). ATF4, activating transcription factor 4; CK2, casein kinase 2.

Human ATF4 is a 351 amino acid long protein organized into two functional domains, an N-terminal transactivation domain (TAD, residues 1–275) and a C-terminal basic-leucine zipper domain (bZip, residues 276–351). The C-terminal bZip domain is composed of a basic region essential for DNA binding (residues 280–301) and leucine zipper region (residues 306–334) essential for homo/heterodimerization (Fig. 1*A*). The bZip domain forms an extended α-helix encompassing both the basic and leucine-zipper regions in a continuous helical structure (13). Like many other transactivation domains in transcription factors, the TAD is predicted to be structurally disordered but plays a key role in modulating the activation and degradation of ATF4 (14). The N-terminal TAD contains a known binding site for several important binding partners, including p300/CBP-associated factor (PCAF) (1) and βTrCP (14, 15, 16). The TAD is also the target of multiple posttranslational modifications. The primary phosphorylation sites of protein kinase A (PKA) (17), ribosomal S6 kinase 2 (RSK2) (18), casein kinase 2 (CK2) (19), and RET tyrosine kinase (20), among others, are all known to be localized within the TAD of ATF4.

Previous NMR studies have shown that a region of TAD encompassing the β-TrCP-binding motif (amino acids 208–230) is disordered in the unphosphorylated state but adopts a hairpin loop structure upon phosphorylation of serines 219 and 224 (15). This phosphorylation-induced conformational change appears to be critical for formation of the β-TrCP-ATF4 complex (16). However, very little is known about the structural features of the full TAD sequence, as an isolated domain or in the context of the full-length ATF4. Furthermore, the mechanisms of interdomain communication and potential coupling with the bZip domain remain unclear. In the present study, we used a combination of bioinformatic sequence analysis, circular dichroism (CD), analytical size-exclusion chromatography coupled with multiangle light scattering (SEC-MALS) and small-angle X-ray scattering (SEC-SAXS), and solution nuclear magnetic resonance (NMR) spectroscopy to determine the structural features of TAD. We found that the transactivation domain is predominantly disordered in solution with the exception of a short stretch of amino acids with a higher helical propensity at the N-terminus of the TAD. In addition, our results show that the TAD and bZip domains are not structurally independent but are coupled through long-range interactions involving the N-terminal region of the TAD. Finally, we found that the presence of the bZip is required to achieve complete phosphorylation of the TAD by the casein kinase CK2, suggesting that coupling between the TAD and bZip domains is essential for optimal posttranslational modification, a crucial mechanism controlling ATF4 proteasomal turnover.

## Results

### ATF4 sequence analysis

We first examined the degree of sequence conservation of ATF4 by comparing the human protein sequence (Uniprot P18848) with those of frog (*Xenopus laevis*, Uniprot Q3V796), chicken (*Gallus gallus*, Uniprot Q9W610), bovine (*Bos taurus*, Uniprot F1MXZ1), goat (*Capra hircus*, Uniprot A0A452EN02), and mouse (*Mus musculus*, Uniprot Q06507). These sequences were aligned using Clustal Omega (21). Figure 1*B* shows the conservation score obtained throughout the sequence and highlights the regions that are highly conserved (score >8 in red) and those that are poorly conserved (score <2 in blue). As expected, the bZip domain, which is essential for DNA binding and dimerization of ATF4, is highly conserved among the compared species (Fig. 1*B*). In contrast, amino acids of the TAD are generally poorly conserved, especially at C-terminus of the transactivation domain. A few notable exceptions include several residues at the N-terminus of the TAD (residues 18, 39, 85, 87, 90, and 95). Analysis of ATF4 amino acid sequence with IDDomainSpotter (22) reveals that the TAD sequence is predominantly enriched in hydrophilic and disorder promoting amino acids (Pro, Ser, and Thr) and depleted in Arg and Lys (represented by positive +PST-RK scores in Fig. 1*C*). Yet, the amino acid composition of two short regions at the N-terminal of the TAD (residues 34–47 and 82–98), which contain conserved amino acids, noticeably differs from the rest of the TAD. Indeed, these regions have negative +PST-RK scores, which indicate a lower propensity for structural disorder (Fig. 1*C*). These differences in amino acid composition within the TAD sequence were confirmed by disorder propensity predictions from DisEMBL (23), PONDR-FIT (24), and DISOPRED (25). Altogether, these predictions show a clear difference in propensity for structural disorder between the N-terminal and C-terminal regions of the TAD. As shown in Figure 1*D*, the first 120 amino acids are indeed predicted to have a significantly lower propensity for structural disorder than amino acids 130 to 275, which are predicted to be almost fully disordered in solution.

### Biophysical characterization of isolated ATF4 TAD

**Figure 2:**
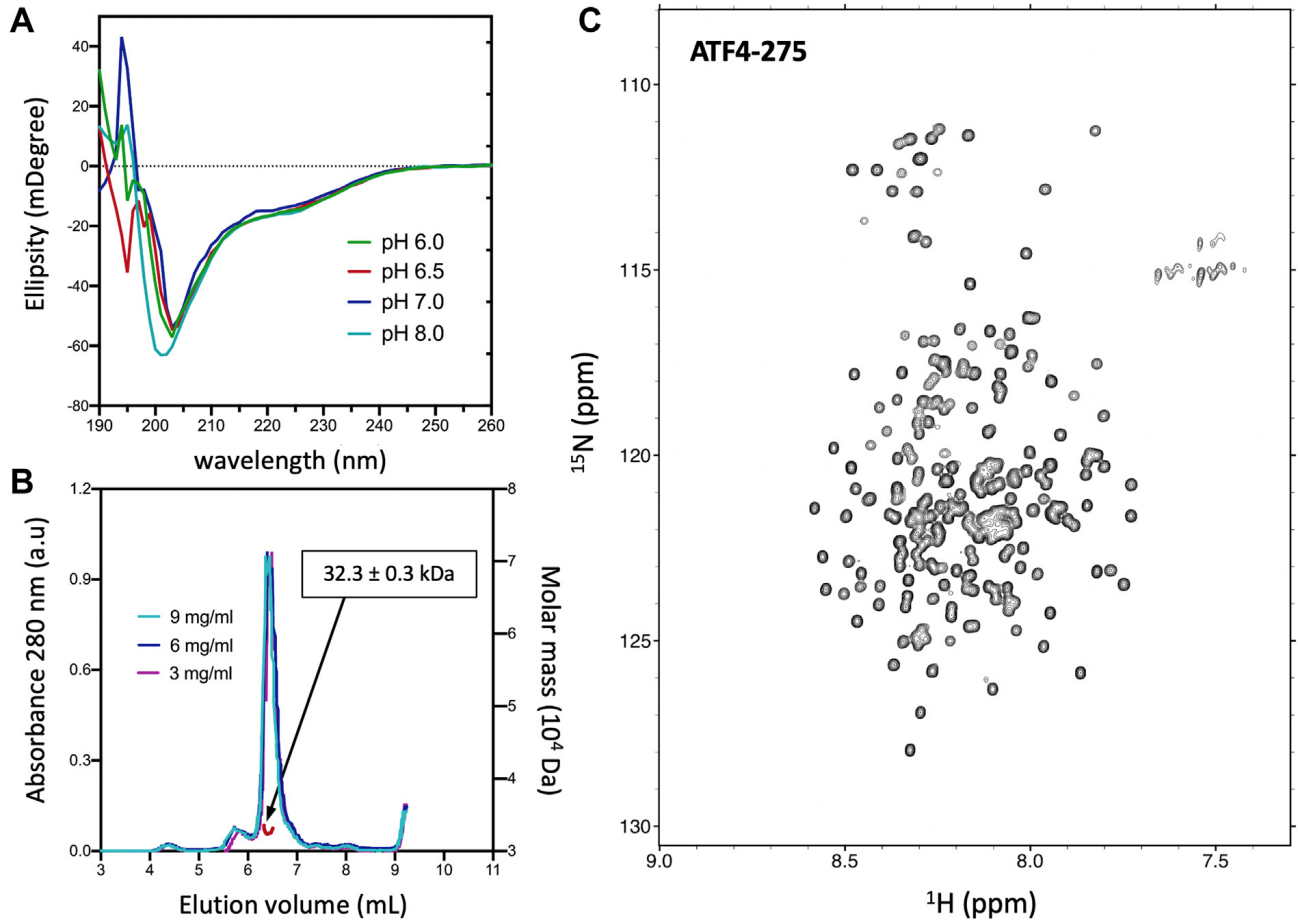
**Biophysical characterization of ATF4 isolated transactivation domain.***A*, CD spectra of ATF4-275 collected at 293 K in four different buffer conditions, with pH ranging from 6.0 to 8.0. *B*, SEC-MALS data showing the elution profiles of ATF4-275 collected at three different protein concentrations, from 3 to 9 mg/ml (*left y-axis*). The MALS profile collected at 9 mg/ml is shown in *red* (*right y-axis*). *C*, 2D ^1^H-^15^N TROSY-HSQC spectrum of ATF4-275 collected in 20 mM MES pH 6.5, 50 mM NaCl, at 293 K. The spectrum was recorded at a ^1^H frequency of 700 MHz. ATF4, activating transcription factor 4; CD circular dichroism; MALS, multiangle light scattering; SEC-MALS, size-exclusion chromatography coupled with multiangle light scattering.

Next, we conducted a range of biophysical experiments to characterize the properties of the isolated transactivation domain of ATF4. We recombinantly expressed and purified a truncated construct of ATF4 encompassing the isolated TAD (ATF4-275, Fig. 1*A*). The CD spectra of ATF4-275 collected at 293 K at a protein concentration of 15 μM, with pH ranging from 6.0 to 8.0, show large negative ellipticity between 200 and 205 nm indicating that the isolated TAD domain is predominantly disordered in solution in all tested conditions (Fig. 2*A*). As shown in Figure 2*B*, the elution profiles of ATF4-275 obtained by SEC-MALS were identical at all three sample concentrations tested (3 mg/ml, 6 mg/ml, and 9 mg/ml) suggesting the absence of oligomerization at higher concentrations. The estimated molecular weight of 32.3 ± 0.3 kDa at 9 mg/mol confirms that the isolated TAD is monomeric in solution (theoretical molecular weight = 29.6 kDa). Additional investigation by SEC-SAXS revealed that ATF4-275 is largely extended in solution, with an Rg of 42.6 ± 4 Å, close to the theoretical dimensions of a fully extended polymeric chain (44 Å) (26) (Fig. S1). We then expressed and purified ATF4-275 in a minimal medium and collected a series of 2D ^1^H-^15^N TROSY-HSQC NMR spectra over a wide range of buffer and pH conditions. We observed in all tested conditions the same narrow ^1^H chemical shift dispersion confirming that ATF4-275 is predominantly disordered in solution (Fig. 2*C*).

### ATF4 TAD possesses a short N-terminal helical motif

Deviations of ^13^C backbone chemical shifts from idealized random coil chemical shifts constitute a powerful method to precisely evaluate the degree of transient secondary structures in intrinsically disordered proteins (27). Yet, assigning the backbone chemical shifts of intrinsically disordered proteins is a challenging task due to the extent of crosspeak overlap. ATF4-275 presents the additional challenges of being a long domain (275 amino acids) with relatively poor sequence complexity (*e.g.*, 32 serines, 32 leucines, 28 prolines). Among all conditions tested, we found that the 2D ^1^H-^15^N spectrum collected in 20 mM MES pH 6.5, 50 mM NaCl presented the most homogeneous crosspeak intensities and minimal crosspeak overlap (Fig. 2*C*). We therefore collected set of conventional 3D triple resonance experiments (HNCA, HNCB, HNCO, HN(CO)CA, and HN(COCA)CB) in this buffer condition. In addition to ATF4-275, we also collected the same set of triple-resonance experiments for a shorter construct encompassing the first 155 amino acids of the TAD (ATF4–155, Fig. 1*A*). This construct was primarily used as a control to check the consistency of our backbone chemical shifts assignment and to resolve potentially ambiguous sequential correlations. Comparison of 2D ^1^H-^15^H spectra collected for ATF4-275 and ATF4-155 shows an almost perfect overlap of the amide crosspeaks, which indicates the absence of long-range contacts between the N- and C-termini of ATF4-275 (Fig. S2). Overall, the set of triple-resonance experiments collected for ATF4-275 and ATF4-155 allowed us to assign the backbone chemical shifts of 254 amino acids (92% of ATF4–275 sequence), with no stretch of unassigned amino acids longer than five consecutive residues (BMRB entry 51062).

**Figure 3:**
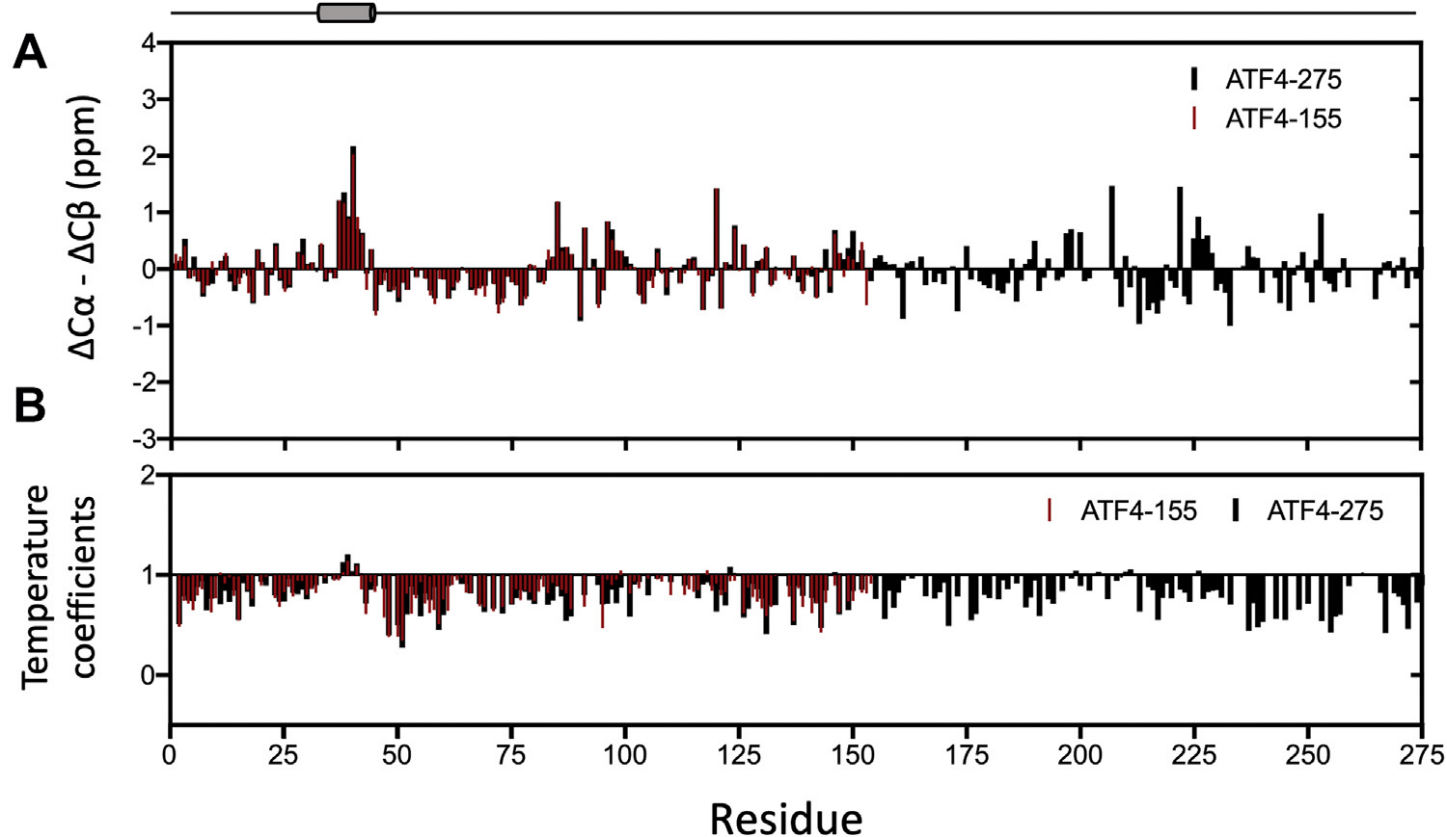
**Solution NMR analysis of ATF4-275 and ATF4-155.***A*, ΔCα-ΔCβ chemical shift deviations shown for ATF4-275 (*black*) and ATF4-155 (*red*) (*B*) Temperature coefficients shown for ATF4-275 (*black*) and ATF4-155 (*red*), calculated as ratios of individual amide crosspeak intensities measured at 293 K and at 283 K (=I_293K_/I_283K_). ATF4, activating transcription factor 4; NMR, nuclear magnetic resonance.

In good agreement with the CD data collected in the same conditions, the overall small ^13^C chemical shift deviations (displayed here as ΔCα- ΔCβ), all within +2 ppm and -1 ppm, confirm that ATF4-275 is predominantly disordered in solution (black in Fig. 3*A*). Nevertheless, upon close examination, a systematic positive chemical shift deviation, indicative of a residual helical motif, is apparent at the N-terminal of the domain (residues 37–44). Strip plots of the HNCA and HN(CO)CA spectra used to solve the sequential assignment of this stretch of amino acids are shown in Fig. S3. The same positive ^13^C chemical shift deviations were observed for these amino acids when measured in the shorter construct ATF4-155 (red in Fig. 3*A*). Beside residues 37 to 44, chemical shift deviations larger than +1 ppm were only found for five residues across TAD sequence. The unique nature of this short helical motif at the N-termini of the TAD is also apparent from temperature coefficients measurement. Here we define these temperature coefficients as ratios between the individual amide crosspeak intensities measured at 293 K and at 283 K (Fig. S4). Because amide protons of intrinsically disordered proteins are largely exposed to solvent, the intensity of amide crosspeaks tends to be very sensitive to changes that would affect the rate of exchange between amide protons and protons from the solvent. Since low temperature limits the rate of exchange with the solvent, amide crosspeaks of disordered proteins are expected to be more intense as the temperature is lowered (temperature coefficients <1). On the other hand, structured regions are expected to be less sensitive to chemical exchange (temperature coefficients ≥1) (28). Figure 3*B* shows that the temperature coefficients measured for ATF4-275 and ATF4-155 are mostly smaller than 1, confirming again that the TAD is predominantly disordered in solution. A notable exception to this trend is found for residues 38 to 44, which exhibit temperature coefficients larger than 1, suggesting that this short motif has a higher structural propensity than the rest of the TAD (Fig. 3*B*).

Overall, these results demonstrate that ATF4 TAD is predominantly disordered in solution but not fully devoid of secondary structure. It encompasses at least one stretch of amino acids at its N-terminus with a higher degree of helical propensity.

### ATF4 TAD domain forms long-range interactions with the bZip domain

**Figure 4:**
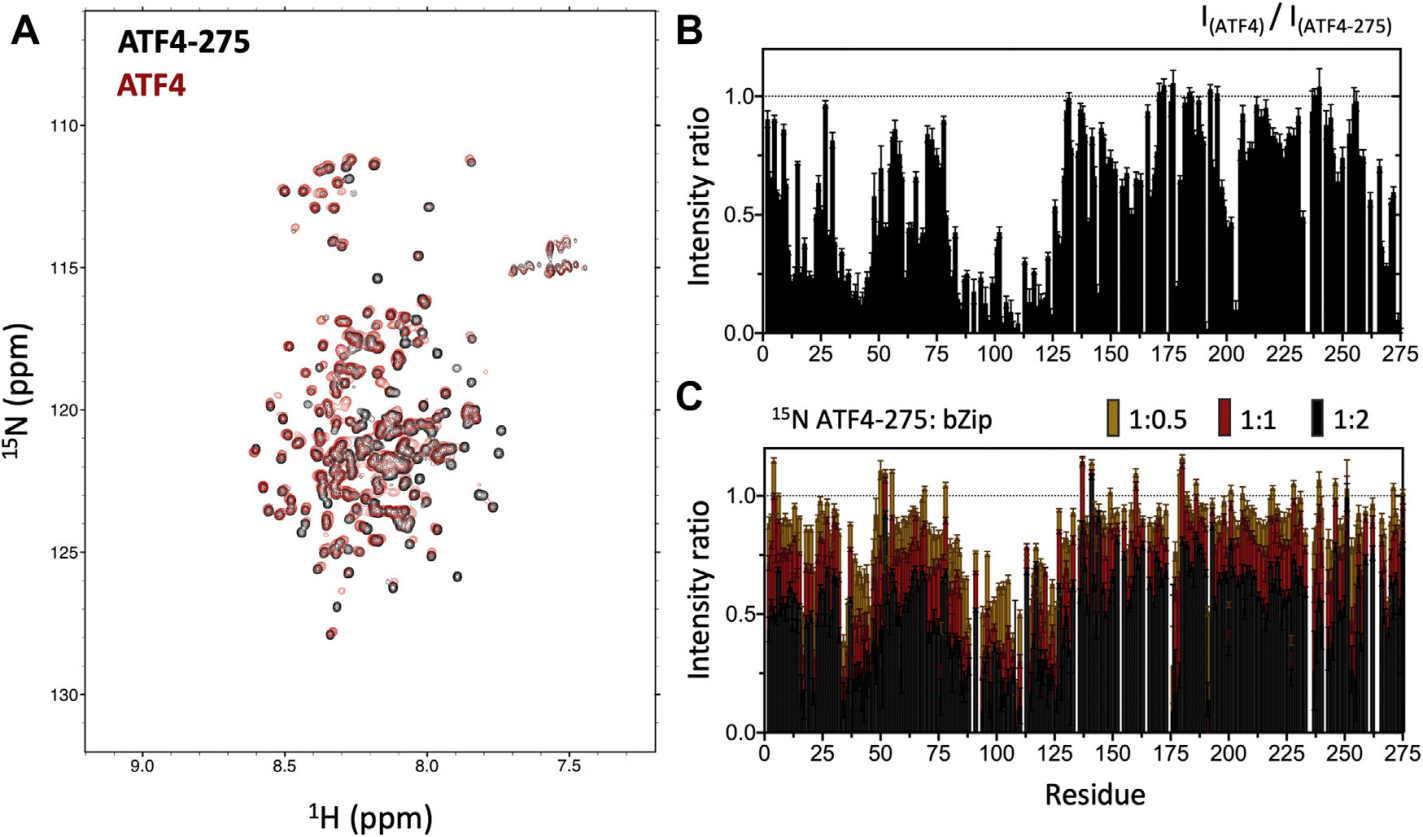
**Comparison of ATF4-275 with the full-length ATF4.***A*, overlay of 2D ^1^H-^15^N TROSY-HSQC spectra collected for ATF4 (*black*) and ATF4-275 (*red*). The spectra were recorded at a ^1^H frequency of 700 MHz. *B*, ratios of individual amide crosspeak intensities measured between ATF4 and ATF4-275 (=I_ATF4_/I_ATF4-275_) (*blue*), and ATF4 with 4 M equivalents of double stranded DNA (*cyan*). *C*, ratios of individual amide crosspeak intensities measured for 2D ^1^H-^15^N TROSY-HSQC spectra of ^15^N-ATF4-275 collected at increasing concentrations of unlabeled ATF4-bZip. ATF4, activating transcription factor 4.

To further study the role played by the transactivation domain in the context of the full-length protein, we compared the NMR spectra of ATF4-275 with that of the full-length ATF4. Besides small chemical shift changes and differences in peak line widths, we observed no additional amide crosspeak that could be assigned to the bZip domain (Figs. 4*A* and S5). This indicates that residues of the bZip domain experience conformational exchange on an intermediate timescale (relative to NMR timescale) leading to excessive line-width broadening. We probed a broad range of buffer and pH conditions, as well as external perturbations (*i.e.*, addition of TMAO and application of high-hydrostatic pressure, data not shown), but despite our efforts, we were unable to shift the timescale of the exchange and/or the relative population of the states in exchange to sufficiently reduce the line-width broadening of bZip resonances.

Another remarkable observation emerging from this comparison is that a significant number of crosspeaks corresponding to TAD residues are drastically attenuated in the full-length ATF4 spectrum (Fig. 4*A*). When plotted in terms of ratios of individual crosspeak intensities between ATF4 and ATF4-275, it clearly appears that theses residues are mostly localized within the N-terminus of the TAD (*e.g.*, residues 13–21, 32–48, and 80–126) (Fig. 4*B*). To assess whether the line-width broadening of these crosspeaks is due to transient interactions between the two domains, we performed a titration of ^15^N-labeled ATF4-275 with unlabeled bZip domain (ATF4-bZip, Fig. 1*A*). We observed upon titration a similar pattern of amide crosspeak attenuation corresponding to residues localized at the N-terminal of the transactivation domain (Figs. 4*C* and S6). Taken together, these NMR results indicate that the TAD and bZip domains are not structurally independent. The excessive line-width broadening appears to result from transient long-range contacts on an intermediate timescale.

### ATF4 bZip domain is required for phosphorylation of the TAD

Finally, we investigated whether the structural coupling between the TAD and bZip domains could impact the ability of the TAD to be posttransitionally modified, which is a key mechanism regulating ATF4 function. The TAD is known to be the target of multiple posttranslational modifications, including phosphorylation by the casein kinase CK2, which controls the proteasomal turnover of ATF4 (14, 19). To test if the presence of the bZip domain influences the ability of TAD to be phosphorylated, we designed a set of *in vitro* phosphorylation assays with CK2 and compared the phosphorylation states of ATF4-275 with that of the full-length ATF4 using solution NMR and mass spectrometry (see Experimental procedures).

**Figure 5:**
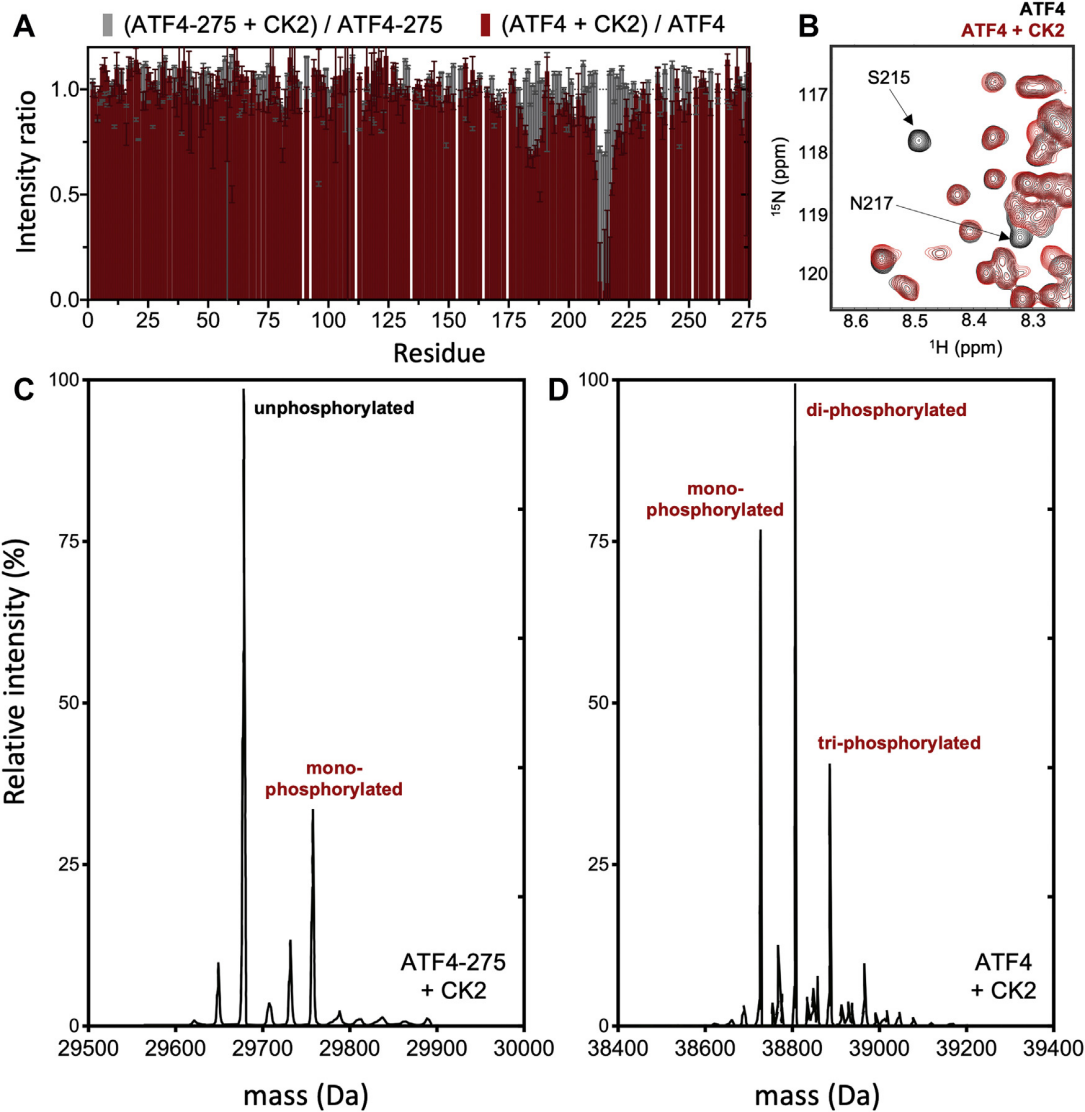
**Comparison of ATF4 and ATF4-275 phosphorylation efficiency by CK2.***A*, ratios calculated as individual amide crosspeak intensities measured between samples incubated with and without the protein kinase CK2 (=I_(+)CK2_)/I_(−)CK2_) for ATF4 (*red*) and ATF4-275 (*gray*). *B*, close-up of 2D ^1^H-^15^N TROSY-HSQC spectra of ATF4 non-incubated with CK2 (*black*) and ATF4 incubated with CK2 (*red*). This region of the spectrum shows the amide crosspeaks of S215 and neighboring residue N217. The spectra were recorded at a ^1^H frequency of 700 MHz. *C*, deconvolution spectrum of intact ATF4-275 after 3 h of incubation with CK2 analyzed by high-performance liquid chromatography coupled to electrospray ionization mass spectrometry (LC-ESI-MS). *D*, deconvolution spectrum of intact ATF4 after 3 h of incubation with CK2. ATF4, activating transcription factor 4; CK2, casein kinase 2.

Comparison of 2D ^1^H-^15^N spectra collected for ATF4 subjected to a 3-h incubation with or without CK2 shows a drastic peak intensity loss for S215 and neighboring residues (red bar in Fig. 5*A*, and red spectrum in Fig. 5*B*), indicating that this serine is almost fully phosphorylated in such conditions by CK2. We also noticed that a second serine (S184) appears to be partially phosphorylated upon incubation with CK2 (Fig. 5*A*). Two new amide crosspeaks are indeed observed in the 2D ^1^H-^15^N spectrum of ATF4 after incubation with CK2: one intense resonance at 8.89 ppm (attributed to the phosphorylated form of S215) and a significantly less intense resonance 8.73 ppm (attributed to the phosphorylated form of S184) (Fig. S7). Interestingly, only minor losses of peak intensity are observed when ATF4-275 is incubated in the same conditions with CK2 (gray bar in Fig. 5*A*), suggesting that phosphorylation by CK2 is significantly less efficient when incubated with the isolated TAD. These results were confirmed by intact protein mass spectrometry showing only partial phosphorylation when CK2 is incubated with ATF4-275 (Fig. 5*C*), while complete phosphorylation is observed with the full-length ATF4, resulting in a distribution of mono, di-, and tri-phosphorylated species (Fig. 5*D* and Table S1). The specific phosphorylation of S215 was confirmed by liquid chromatography with tandem mass spectrometry (LC-MS/MS) (Table S2). Overall, these results suggest that the presence of the bZip domain is essential for *in vitro* phosphorylation of TAD by the kinase CK2.

## Discussion

Nearly 90% of eukaryotic transcription factors contain large transactivation domains that are intrinsically disordered (29). These TADs can form long continuous domains up to several hundred residues or be dispersed through different regions of the protein, as in the case of p53 (30). As regulatory hubs for transcriptional coactivators and repressors, TADs play an essential role in modulating the activity of transcription factors (31, 32). TADs are also usually heavily posttranscriptionally modified, which adds an extra layer of complexity and modularity to their ability to control transcriptional activity (33, 34). Being predominantly disordered in solution, TADs are believed to exert their functions through relativity weak, highly dynamics, and short-lived interactions (35). While critically important, the structural and mechanistic basis of TAD functions is still poorly understood, in part due to the major challenges inherent to characterizing disordered domains. Biophysical approaches able to identify and characterize structured motifs at a residue level within a predominantly disordered conformational ensemble are essential to fully understand the mechanisms of regulation of transcription factors.

In the present study, we used a range of biophysical techniques to characterize the residual structural propensity of ATF4 TAD, a transcription factor that plays a key role in mediating the expression of a variety of genes in response to cellular stresses. Analysis of ATF4 sequence highlights the differences between the N-and C-terminal regions of the TAD. The first 125 amino acids of the TAD appear to have characteristics closer to that of a folded-like region rather than of a typical intrinsically disordered region. Indeed, we found that residues 1 to 125 are overall more conserved, less enriched in disorder-promoting amino acids, and have a relatively low propensity for structural disorder compared with residues 126 to 275 (Fig. 1, *B*–*D*). Differences between the N- and C-terminal regions of ATF4 TAD have been previously proposed by Liang and Hai (1), who identified residues 91 to 124 as a potential additional leucine zipper motif.

Here, a combination of CD, SEC-MALS, SEC-SAXS, and NMR experiments unambiguously show that the isolated TAD is predominantly disordered in solution (Fig. 2), suggesting that the more foldable characteristics of the first 125 amino acids are not sufficient to significantly shift the conformational ensemble of the TAD toward a folded-like ensemble with stable secondary structures. Yet, backbone chemical shift analysis, complemented by temperature coefficients, revealed that the N-terminal region is not fully disordered in solution but contains at least one short motif (residues 37–44) with high helical propensity (Fig. 3). Importantly, we found that most of the N-terminal region (*i.e.*, residues 13–21, 32–48, and 80–126) appears to be involved in transient long-range interactions with the bZip domain on an intermediate timescale (Fig. 4). While this type of coupling between the TAD and bZip domains have, to the best of our knowledge, not been identified before for ATF4, it is strongly reminiscent of other transcription factors such as p53 (36) and FOXO4 (37). In both cases, regions of the transactivation domains are known to interact directly with the DNA-binding domain (36, 37). Intrinsically disordered domains can mediate interactions through a wide range of mechanisms, from induced fit (38) to conformational selection (39), and various mixed mechanisms (40, 41). In the case of ATF4, the higher helical propensity of residues at the N-terminus of TAD suggests that interaction between the TAD and bZip domains takes place through conformational selection. However, precise kinetic information will be required to unequivocally determine the exact binding mechanism.

Finally, using a combination of NMR and mass spectrometry experiments, we found that the presence of the bZip domain is required for full phosphorylation of TAD by the kinase CK2. Among all 12 putative sites in ATF4 with the consensus sequence S/TXXD/E, S215 has been shown previously to be primary phosphorylation site of CK2, both *in vitro* and *in vivo* (19). Here, we found that incubation with CK2 led to complete phosphorylation of S215 in the full-length ATF4 but not in the isolated TAD (Fig. 5). This observation can be explained in several ways: (i) access of the phosphorylation motif to CK2 active site is facilitated by the disorder-mediated coupling between the two domains, (ii) CK2 binds to bZip, triggering a conformational change that brings the phosphorylation motif within reach, or (iii) CK2 optimal binding site covers regions of both TAD and bZip domains. Characterization of single point variants of the bZip domain is currently ongoing in our group in an effort to determine the precise mechanism of phosphorylation of ATF4 by CK2.

While it is well documented that TADs can modulate the accessibility of DNA-binding domains to limit or enhance DNA binding (36, 37, 42), our results suggest that this type of fine regulation may be reciprocal, as in the case of ATF4, the bZip domain modulating the accessibility of the TAD to a protein kinase. Altogether, these findings shed light on the complexity and intricate nature of these disorder-driven activation mechanisms that may be shared by many eukaryotic transcription factors.

## Experimental procedures

### Sequence analysis

Clustal Omega (https://www.ebi.ac.uk/Tools/msa/clustalo/) was used with default parameters. +PST-RK scores were calculated using IDDomainSpotter (http://www2.bio.ku.dk/IDR/) with 20 amino acids window. Disorder propensity calculated with DisEMBL (http://dis.embl.de/) is reported here for the “coils” values with coils threshold set to 1.20. The predictor VL3 was used with *via* PONDR-FIT (http://original.disprot.org/pondr-fit.php). The algorithm Disopred3 was used *via* PSIPRED (http://bioinf.cs.ucl.ac.uk/psipred/).

### Sample preparation

cDNA fragments encoding ATF4, ATF4-155, ATF4-275, and ATF4-bZip sequences were amplified by PCR and inserted into pGEX-4T1 plasmid using EcoRI and XhoI restriction sites. All constructs contained an N-terminal glutathione S-transferase (GST) followed by TEV protease cleavage site. Expression was performed in *E. coli* BL21(DE3) cells. Cells were grown at 37 °C overnight. The next day, 10 ml of overnight culture was transferred to 1 L of fresh medium and grown at 37 °C until OD_600nm_= 0.8. Protein overexpression was induced by adding IPTG to a final concentration of 400 μM and shaking at 16 °C overnight. Cells were harvested by centrifugation at 6000 rpm. Cells overexpressing ATF4, ATF4-275, and ATF4-155 were resuspended in Lysing Buffer A (50 mM Tris-HCl pH 8.0, 100 mM NaCl, 5 mM DTT, 1 mM EDTA). Cells overexpressing ATF4-bZip were resuspended in Lysing Buffer B (50 mM CHES 9.0, 100 mM NaCl, 5 mM DTT, 1 mM EDTA). Both lysing buffers were supplemented with PMSF, Leupeptin, DNase I, and Protease Inhibitor Cocktails (Alpha Aesar). Cells were lysed by sonicating at 4 °C. The insoluble fractions were removed by centrifugation at 16,000 rpm for 1 h at 4 °C. GST-tagged proteins were purified by passing the supernatant through Glutathione-Sepharose beads (UBPBio). Unbound proteins were removed by washing with lysing buffer, and GST-tagged proteins were eluted with lysing buffers containing 5 mM reduced glutathione. Additionally, ATF4 and ATF4-bZip were washed with 50 mM Tris-HCl 8.0, 500 mM NaCl, 5 mM DTT, 1 mM EDTA to remove nonspecific bound nucleic acids. After incubating with 0.01 mg of TEV protease per mg of GST-tagged ATF4, ATF4-155, and ATF4-275 for 1 h, the cleaved GST-tags were removed by running through two subtractive GST affinity columns. The GST-tagged ATF4-bZip was incubated with 0.01 mg of TEV protease overnight (∼16 h), and the cleaved GST-tag and ATF4-bZip were then separated by running through a HiLoad 16/600 SuperdexTM 200 pg gel filtration column in SEC Buffer (20 mM MES 6.5, 50 mM NaCl). The samples were maintained at 4 °C throughout the purification process.

### Circular dichroism

Circular dichroism (CD) spectra were recorded using an MOS-500 (Biologic) spectropolarimeter controlled with Bio-Kine32 at room temperature in a 1 mm UV quartz cuvette (FireflySci). Samples were prepared at 15 μM in the following buffers: (i) 20 mM MES pH 6.0, 50 mM NaCl, (ii) 20 mM MES pH 6.5, 50 mM NaCl, (iii) 20 mM HEPES pH 7.0, 50 mM NaCl, and (iv) 20 mM Tris-HCl pH 8.0, 50 mM NaCl. The mean spectra were derived from three acquisitions collected between 190 –and 260 nm for 0.2 s with 2 nm slit width. Spectra were background-corrected with buffers.

### SEC-MALS and SEC-SAXS

Multiangle light scattering (MALS) and small-angle X-ray scattering (SAXS) coupled in-line with size-exclusion chromatography (SEC) experiments were performed at SIBYLS ALS beamline 12.3.1 LBNL. SEC-MALS and SEC-SAXS experiments were performed with 70  μl samples of ATF4-275 prepared at 3 mg/ml, 6 mg/ml, and 9 mg/ml (∼100 μΜ, 200 μΜ, 300 μΜ protein concentration). The X-ray wavelength was set at λ = 1.03 Å incident light at a sample to detector distances of 1.5 m resulting in scattering vectors (q) ranging from 0.013 to 0.5 Å^−1^. The scattering vector is defined as q = 4πsinθ/λ, where 2θ is the scattering angle. All experiments were performed at 20  °C, and the data were processed as described previously (43). Briefly, an SAXS flow cell was directly coupled with an online 1260 Infinity HPLC system (Agilent) using a Shodex 803 analytical column (Showa Denko) for SEC separation. The column was equilibrated with a running buffer (20 mM MES pH 6.5, 50 mM NaCl) with a flow rate of 0.5 ml/min. Sixty-five microliters of each sample was run through the SEC, and 3-s X-ray exposures were collected continuously during a 40-min elution. The SAXS frames recorded prior to the protein elution peak were used to subtract all other frames. Final merged SAXS profiles, derived by integrating multiple frames across the elution peak, were used for further analysis using PRIMUS (44) from the ATSAS suite of programs (45).

Eluent was subsequently split into 2:1 between the SAXS line and a series of UV at 280 and 260 nm detectors, quasi-elastic light scattering (QELS) detectors, and MALS detectors. MALS experiments were performed using a Wyatt Dawn Heleos MALS detector connected in tandem to an Optilab rEX refractive index concentration detector (Wyatt Technology). The light-scattering experiments were used to perform analytical scale chromatographic separations for molecular weight determination of the principal peaks in the SEC analysis.

### Solution NMR spectroscopy

NMR spectra were collected on a Bruker 700 MHz and 800 MHz spectrometers, equipped with z-shielded gradient triple resonance 5 mm TCI cryoprobe. NMR samples were prepared at protein concentrations of 180 to 220 μM in 20 mM MES pH 6.5, 50 mM NaCl, and 90% H_2_O/10% D_2_O (v/v). 2D ^1^H-^15^N TROSY-HSQC experiments were recorded with a time domain matrix consisting of 100∗ (t_1_, ^15^N) × 1024∗ (t_2_, ^1^H) complex points with acquisition time of 50 ms (t_1_) and 91.8 ms (t_2_) using eight scans per FID and 1.5 s interscan delay. Spectral widths for 1H and 15N dimensions were set to 15.9 and 28.2 ppm, respectively, with carriers set at 4.821 ppm (^1^H) and 119.138 ppm (^15^N). Sequential ^1^H/^15^N/^13^C backbone assignments of ATF4-275 and ATF4-155 were achieved using TROSY versions of conventional 3D triple resonance correlation experiments [HNCO, HNCA, HNCACB, HN(CO)CA, and HN(COCA)CB] (46) at 700 MHz. Secondary ^13^C chemical shifts were calculated using Poulsen’s random coil database with corrections for pH, temperature, and neighbor amino acid correction (47, 48). All spectra were processed using NMRPipe (49) and displayed with SPARKY (50).

### *In vitro* phosphorylation

ATF4 and ATF4-275 constructs were phosphorylated in kinase buffer (20 mM HEPES pH 7.5, 50 mM NaCl, 10 mM MgCl_2_, and 1 mM DTT). The phosphorylation reactions were started by adding 2.5 mM ATP and 25,000 units of CK2 (two alpha and two beta subunits, NEB) per mg of protein sample. The phosphorylation reactions were performed for 3 h at 25 °C. The reactions were quenched by buffer exchange (*i.e.*, three rounds of centrifugal concentration (3K MWCO) with intervening buffer dilutions) to remove ATP and MgCl_2_.

### Intact protein mass spectrometry

Liquid chromatography–mass spectrometry (LC-MS) on intact protein samples was performed using an ACQUITY UPLC H-Class coupled in line to a ESI-Q-TOF Synapt G2-Si (Waters) using a Restek Ultra C4 5 μm (#9102551) reverse-phase column. The column was operated at 25 °C with the flow rate of 0.4 ml/min. Chromatography was performed with a linear gradient between mobile phases A and B, with Buffer A consisting of aqueous 0.1% formic acid and Buffer B consisting of 100% acetonitrile, 0.1% formic acid. The elution was carried out using a linear gradient from 5% to 100% B at a flow rate of 0.4 ml/min for 7 min. Protein samples were injected at a concentration of 0.1 mg/ml. Mass spectrometry was performed using a LockSpray ESI (Waters) ionization source with [Glu1]-fibrinopeptide B as internal lock mass calibrant. The instrument was operated using MassLynx 4.1 (Waters). MS data were collected in positive ion, MS continuum, resolution mode with an m/z range of 300 to 5000. The intact protein molecular weight of each sample was deconvoluted from multiple charge states using the MaxEnt3 function of MassLynx 4.2 (Waters).

### Liquid chromatography–tandem mass spectrometry

Phosphorylated samples were alkylated with 15 mM of iodoacetamide at 25 °C for 30 min before digesting with chymotrypsin at 25 °C for 20 h followed by trypsin at 37 °C for 10 h. Digested peptides were injected into EASY nLC-1200 coupled to a Nanospray FlexIon source for in-line analysis with a Q Exactive Hybrid Quadrupole-Orbitrap Mass Spectrometer equipped with an HCD fragmentation cell (Thermo Scientific). Peptides were searched with a Mascot database containing the GST-tagged ATF4 sequence. Peptides were identified with Proteome Discoverer Software 2.4 using the following search parameters: false discovery rate (FDR) 1%, precursor mass tolerance 20 ppm, and fragment mass tolerance ± 0.02 Da. Oxidation (M), deamidation (NQ), and phosphorylation (ST) were searched as dynamic modifications, and carbamidomethylation (C) was set as a static modification.

## Data availability

The backbone chemical resonance assignment of ATF4-275 has been deposited at the Biological Magnetic Resonance Data Bank (BMRB) under accession number 51062. The mass spectrometry raw data and results are deposited to PRIDE Submission under accession number PXD029704.

## Supporting information

This article contains supporting information.

## Conflict of interest

The authors declare that they have no conflicts of interest with the contents of this article.
