# Supplementary material for: Intricate coupling between the transactivation and basic-leucine zipper domains governs phosphorylation of transcription factor ATF4 by casein kinase 2
Source: J Biol Chem. 2022 Jan 22;298(3):101633. doi: 10.1016/j.jbc.2022.101633 (PMC8881488; doi:10.1016/j.jbc.2022.101633)
Supplement: Supplemental Figures S1–S7, Table S1 and S2 [file mmc1.docx]

Supporting information for:

Intricate coupling between the transactivation and basic-leucine zipper domains governs phosphorylation of transcription factor ATF4 by casein kinase 2

Steven Siang, Eric S. Underbakke, and Julien Roche*

Roy J. Carver Department of Biochemistry, Biophysics and Molecular Biology, Iowa State University, Ames, IA 50011, United States.


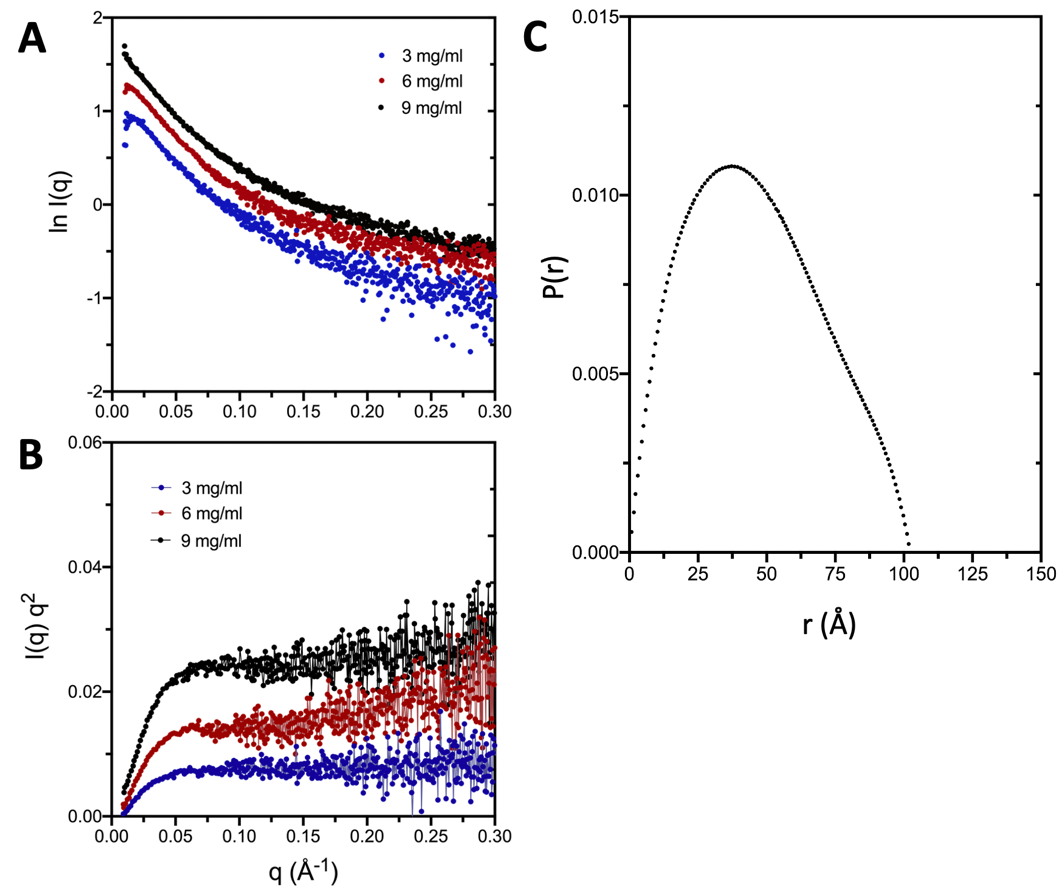


**Figure S1**. Small-angle X-ray scattering (SAXS) profile of ATF4-275 collected by SEC-SAXS experiments. (**A**) Raw intensity profiles at three concentrations: 3 mg/ml (blue), 6 mg/ml (red), and 9 mg/ml (yellow. (**B**) Kratky plots showing plateaued baselines at high q values indicating that ATF4-275 is predominately disordered in solution at all three concentrations. (**C**) Pair-wise distance distribution function P(r) calculated for the highest concentration data set (9 mg/ml).


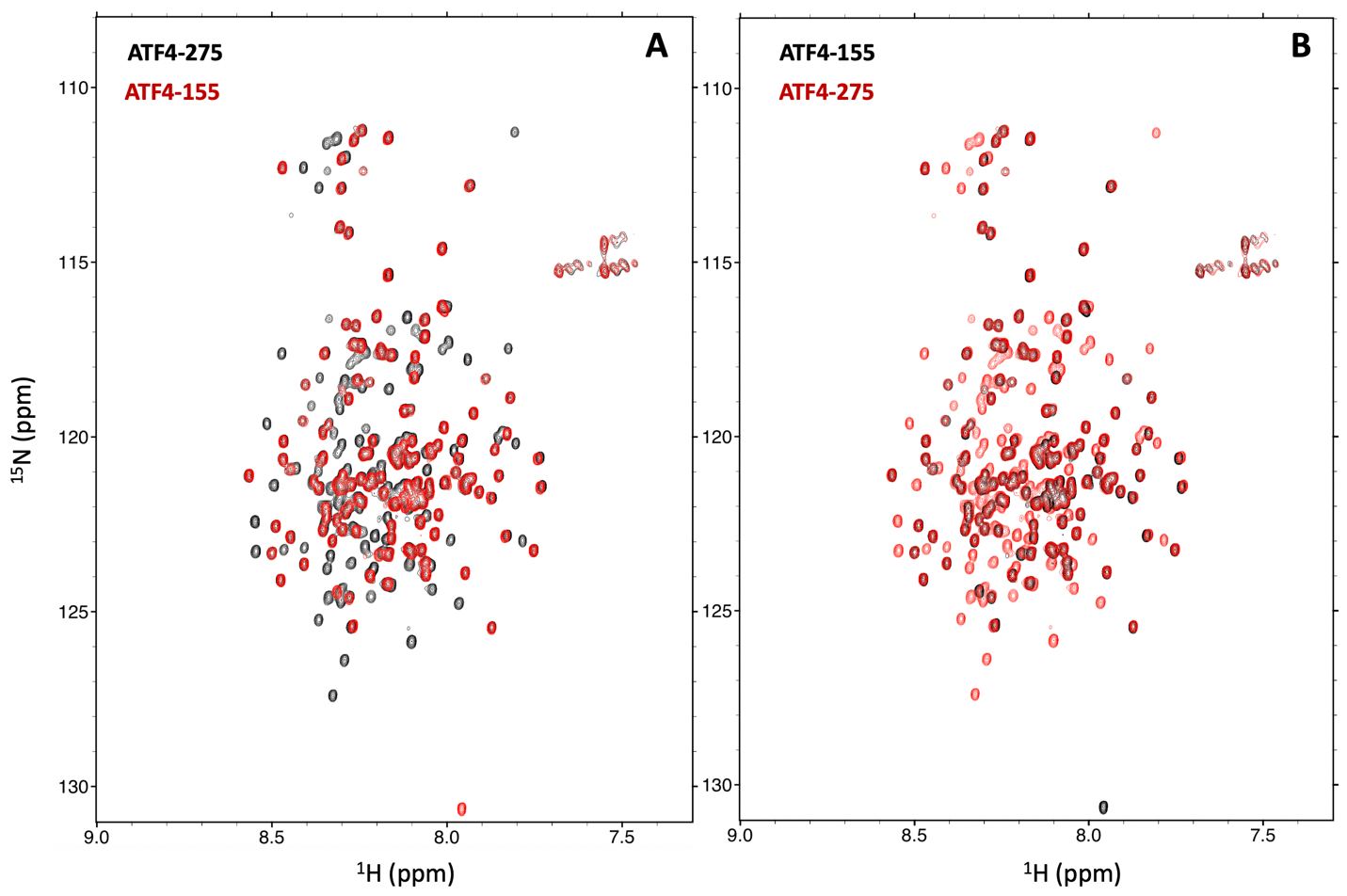


**Figure S2**. Overlay of 2D ^1^H-^15^N TROSY-HSQC spectra collected for ATF4-275 and ATF4-155 at pH 6.5, 293 K. (**A**) ATF4-275 in the background (in black) and ATF4-155 in the foreground (in red). (**B**) ATF4-155 in the background (in black) and ATF4-275 in the foreground (in red). The spectra were recorded at a ^1^H frequency of 700 MHz


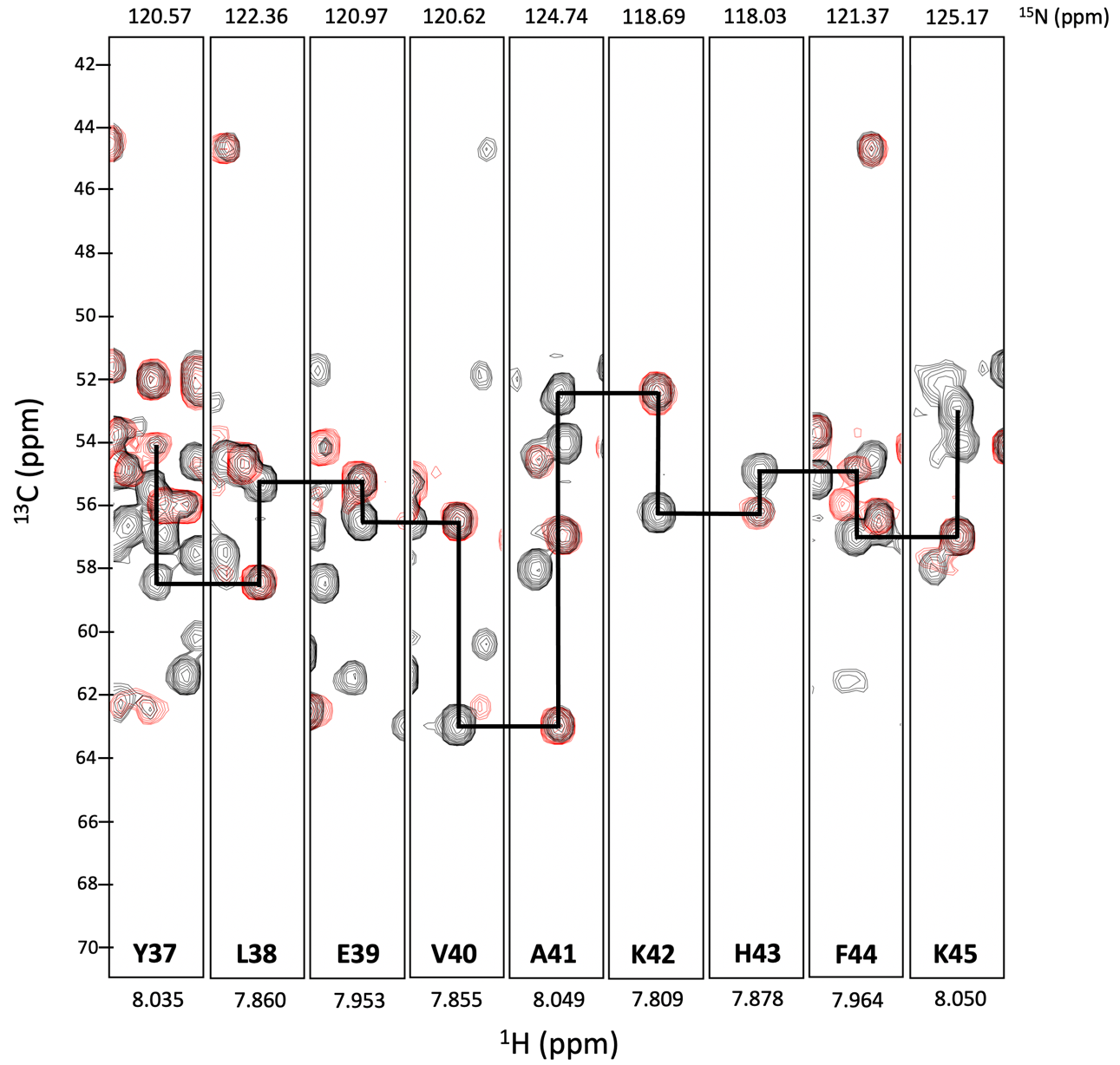


**Figure S3**. Overlay of the strip plots obtained from the HNCA (in black) and HN(CO)CA spectra measured for ATF4-275 at pH 6.5, 293 K. The black line highlights the sequential assignment of residues 37 to 45, which show the highest helical propensity within TAD sequence. The spectra were recorded at a ^1^H frequency of 700 MHz.


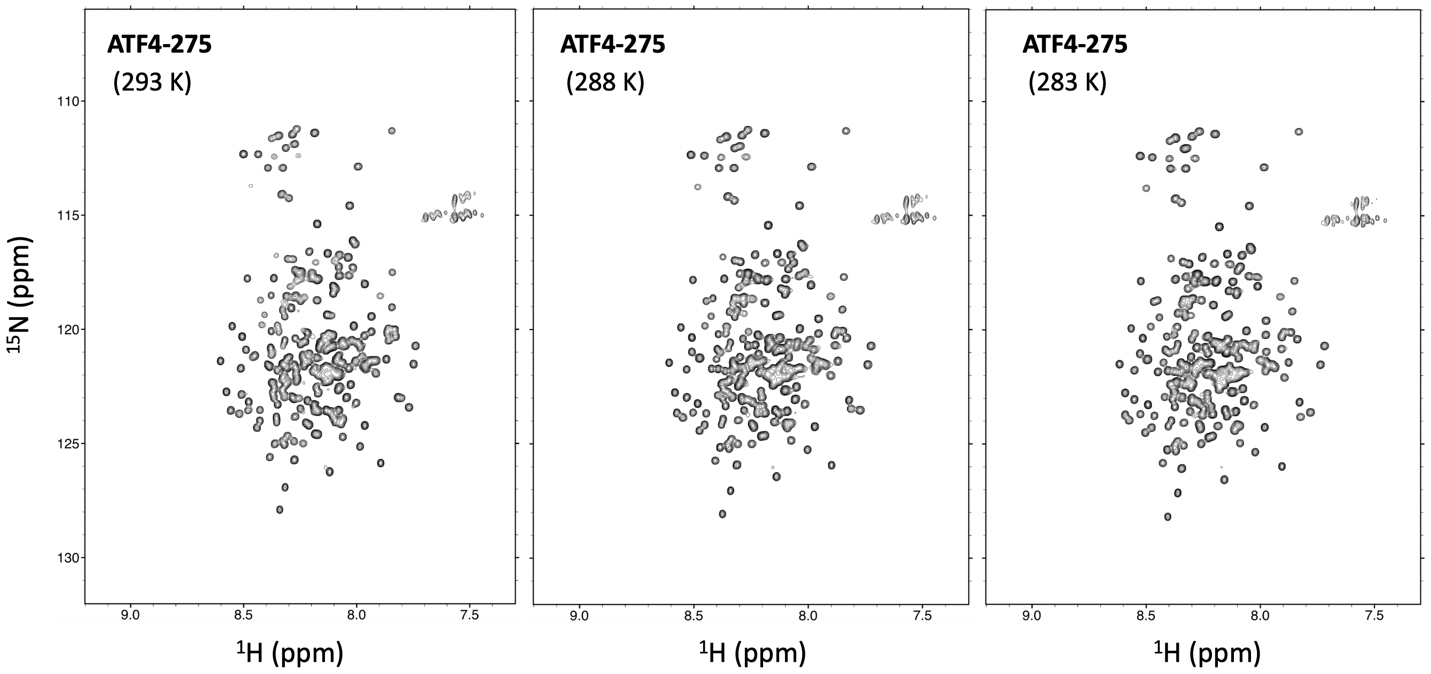


**Figure S4**. 2D ^1^H-^15^N TROSY-HSQC spectra collected for ATF4-275 at pH 6.5 and various temperatures: (i) 293 K (left), (ii) 288 K (center), and (iii) 283 K (right). The spectra recorded at a ^1^H frequency of 700 MHz.


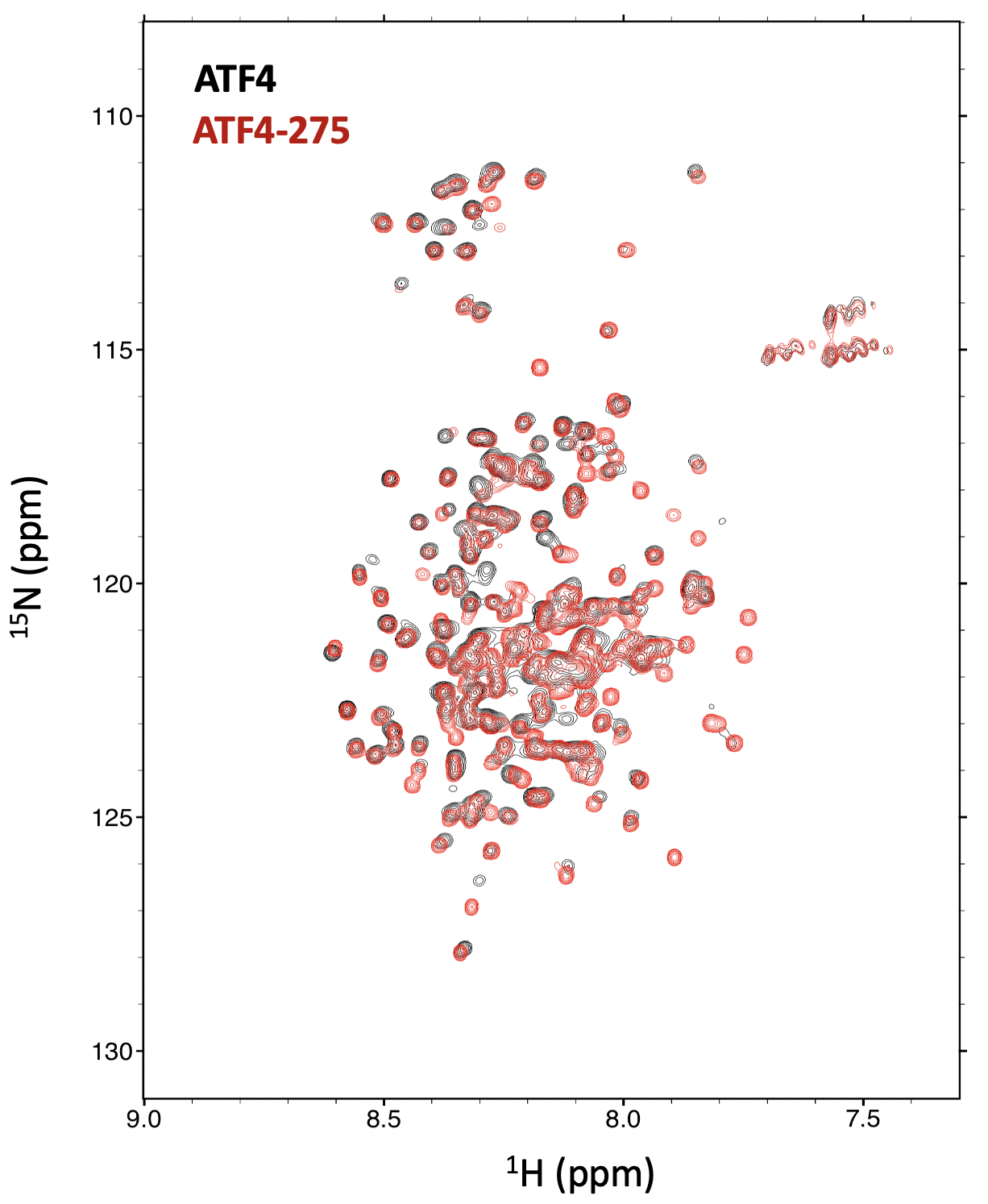


**Figure S5**. Overlay of 2D ^1^H-^15^N TROSY-HSQC spectra collected for ATF4 (in black) and ATF4-275 (in red) at pH 6.5, 293 K. The spectra were recorded at a ^1^H frequency of 700 MHz.

**
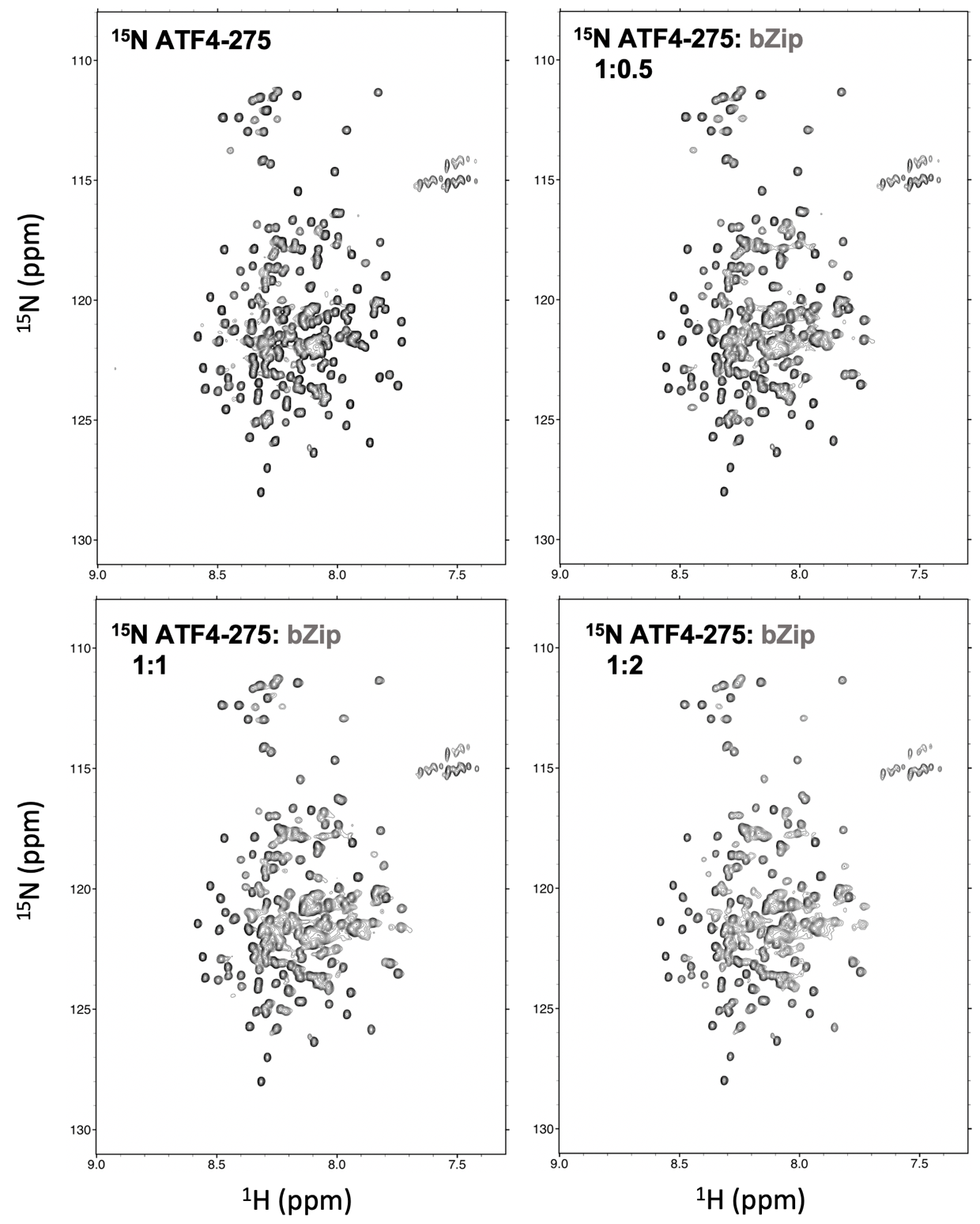
**

**Figure S6**. Series of 2D ^1^H-^15^N TROSY-HSQC spectra collected at pH 6.5, 293 K upon titration of ^15^N-labeled ATF4-275 with increasing concentrations of unlabeled ATF4-bZip. The spectra were recorded at a ^1^H frequency of 800 MHz.

**
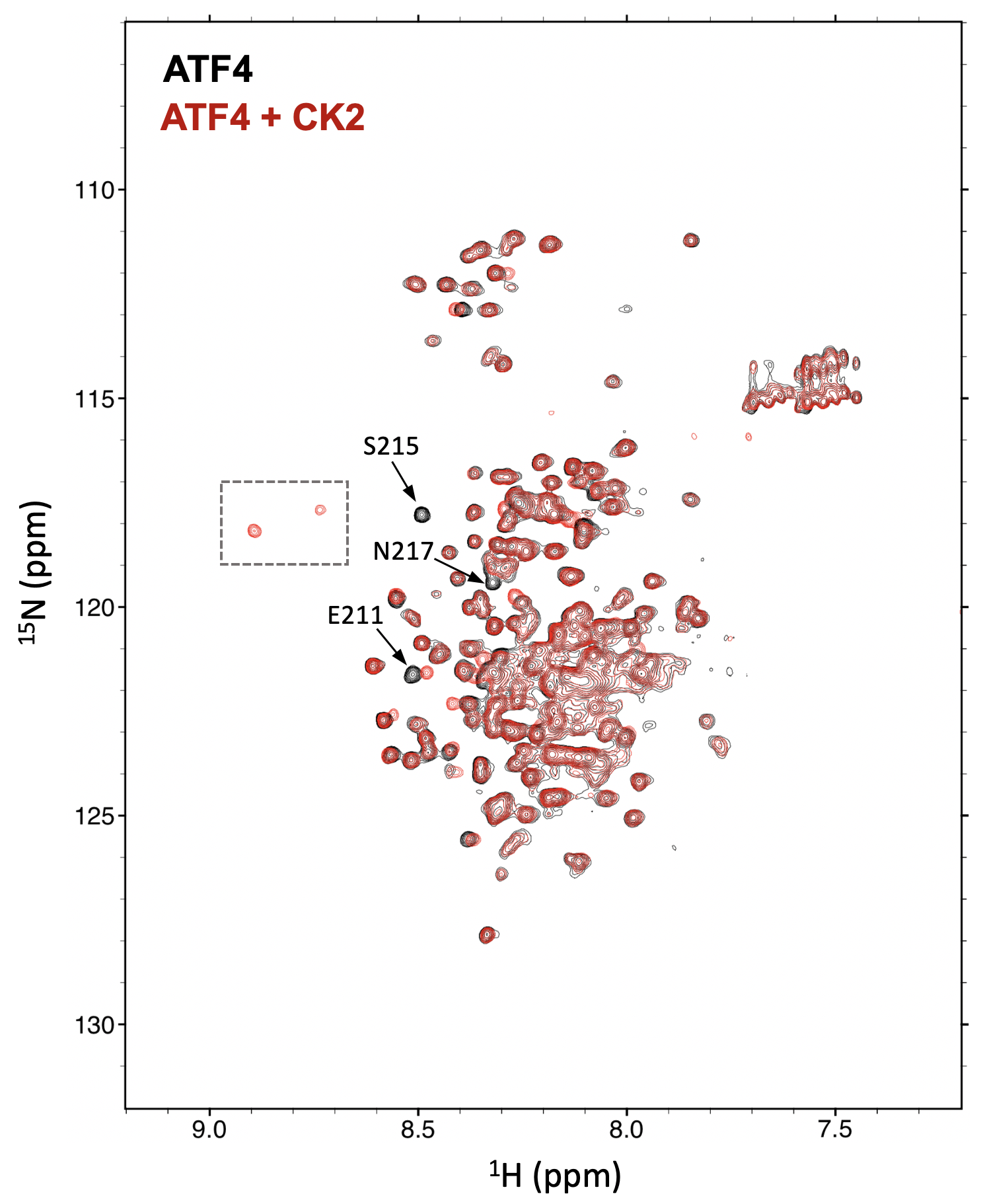
**

**Figure S7**. Overlay of 2D ^1^H-^15^N TROSY-HSQC spectra collected at pH 6.5, 293 K of ATF4 in the absence of CK2 (in black) and after a 3 hour-long incubation with CK2 (in red). The dashed box highlights the presence of two new amide crosspeaks that appeared after incubation with CK2. The spectra were recorded at a ^1^H frequency of 700 MHz.

| **Protein** | **Expected Mass**  **(Da)** | **Observed Mass**  **(Da)** | **Mass Error**  **(ppm)** |
| --- | --- | --- | --- |
| mono-phosphorylated ATF4 | 38,726 | 38,727 | 26 |
| di-phosphorylated ATF4 | 38,806 | 38,807 | 26 |
| tri-phosphorylated ATF4 | 38,886 | 38,886 | 0 |
| mono-phosphorylated ATF4-275 | 29,678 | 29,678 | 0 |
| mono-phosphorylated ATF4-275 | 29,758 | 29,758 | 0 |

**Table S1. Comparison between expected and observed masses from Intact Protein Mass Spectrometry.** The protein average molecular mass was determined by deconvoluting the m/z of multiple charge states using MassLynx 4.2 with MaxEnt3 (Waters).

| **Sequence**  **number** | **Peptide** | **Modification** | **Ion**  **Score** | **Ion Relative**  **Abundance** |
| --- | --- | --- | --- | --- |
| 21-31 | DQSGLGAEESL | 1xPhospho [S3] | 57 | 1.0E+07 |
| 62-75 | LAVDGLVSPSNNSK | 1xPhospho [S8] | 93 | 3.0E+07 |
| 76-84 | EDAFSGTDW | 1xPhospho [T7] | 15 | 2.2E+07 |
| 101-113 | GIDDLETMPDDLL | 1xPhospho [T7] | 39 | 9.8E+06 |
| 171-178 | SSTPDHSF | 1xPhospho [S/T] | 24 | 4.2E+07 |
| 166-177 | SPGVLSSTPDHSF | 1xPhospho [S6] | 67 | 7.1E+08 |
| 179-193 | SLELGSEVDITEGDR | 1xPhospho [S6] | 124 | 1.3E+09 |
| 181-193 | ELGSEVDITEGDR | 1xPhospho [S4] | 80 | 1.3E+08 |
| 209-228 | EEDTPSDNDSGICMSPESY | 1xPhospho [T/S] | 24 | 2.2E+08 |
| 209-228 | EEDTPSDNDSGICMSPESY | 1xPhospho [S6] | 21 | 1.1E+08 |
| 300-311 | RAEQEALTGECK | 1xPhospho [T8] | 77 | 9.4E+06 |

**Table S2. Primary phosphorylation sites of full-length ATF4.** ATF peptides derived from sequential chymotrypsin and trypsin digestion were injected into EASY nLC-1200 coupled to a Nanospray FlexIon source for in-line analysis with a Q Exactive Hybrid Quadrupole-Orbitrap Mass Spectrometer equipped with an HCD fragmentation cell (Thermo Scientific). Peptides were identified with Proteome Discoverer™ Software 2.4 using the following search parameters: false discovery rate (FDR) 1% , precursor mass tolerance 20 ppm, and fragment mass tolerance +/- 0.02 Da.
